# Denosumab treatment and infection risks in patients with osteoporosis: propensity score matching analysis of a national-wide population-based cohort study

**DOI:** 10.3389/fendo.2023.1182753

**Published:** 2023-05-19

**Authors:** Shih-Ting Huang, Ting-Fang Chiu, Chih-Wei Chiu, Yu-Nong Kao, I-Kang Wang, Chi-Tzung Chang, Chi-Yuan Li, Chung-Shu Sun, Cheng-Li Lin, Tung-Min Yu, Chia-Hung Kao

**Affiliations:** ^1^ Division of Nephrology, Taichung Veterans General Hospital, Taichung, Taiwan; ^2^ Graduate Institute of Public Health, China Medical University, Taichung, Taiwan; ^3^ Department of Internal Medicine, College of Medicine, China Medical University, Taichung, Taiwan; ^4^ Department of Post-Baccalaureate Medicine, College of Medicine, National Chung Hsing University, Taichung, Taiwan; ^5^ Department of Pediatrics, Taipei City Hospital Zhongxiao Branch, Taipei, Taiwan; ^6^ Department of Health and Welfare, University of Taipei, Taipei, Taiwan; ^7^ School of Medicine, National Yang Ming Chiao Tung University, Taipei, Taiwan; ^8^ Graduate Institute of Business Administration, Fu Jen Catholic University, New Taipei, Taiwan; ^9^ Department of Nephrology, Kaohsiung Medical University Baccalaureate Medicine, Kaohsiung, Taiwan; ^10^ Graduate Institute of Biomedical Sciences and School of Medicine, College of Medicine, China Medical University, Taichung, Taiwan; ^11^ Management Office for Health Data, China Medical University Hospital, Taichung, Taiwan; ^12^ Department of Bioinformatics and Medical Engineering, Asia University, Taichung, Taiwan; ^13^ Department of Nuclear Medicine and PET Center, China Medical University Hospital, Taichung, Taiwan; ^14^ Artificial Intelligence Center, China Medical University Hospital, Taichung, Taiwan

**Keywords:** denosumab, immunity, RANKL inhibition, T-cell, osteoporosis

## Abstract

**Introduction:**

Denosumab demonstrates efficacy in reducing the incidence of hip, vertebral, and nonvertebral fractures in postmenopausal women with osteoporosis. We present a population-based national cohort study to evaluate the infection risks in patients with osteoporosis after long-term denosumab therapy.

**Methods:**

We used the Taiwan National Health Insurance Research Database (NHIRD) to identify patients with osteoporosis. The case cohort comprised patients treated with denosumab. Propensity score (PS) matching was used to select denosumab nonusers for the control cohort. The study period was between August 2011 and December 2017. Our study comprised 30,106 pairs of case and control patients.

**Results:**

Patients receiving denosumab therapy had high risks of the following infections: pneumonia and influenza (adjusted hazard ratio [aHR]: 1.33; 95% confidence interval [CI]: 1.27 -1.39), urinary tract infection (aHR: 1.36; 95% CI:1.32 -1.40), tuberculosis (aHR: 1.60; 95% CI: 1.36 -1.87), fungal infection (aHR: 1.67; 95% CI:1.46 -1.90), candidiasis (aHR: 1.68; 95% CI: 1.47 -1.93), herpes zoster infection (aHR: 1.27; 95% CI: 1.19 -1.35), sepsis (aHR: 1.54; 95% CI:1.43 -1.66), and death (aHR: 1.26; 95% CI: 1.20 -1.32). However, the longer the duration of denosumab treatment, the lower the risk patients had of developing infections.

**Discussion:**

Denosumab therapy is associated with a higher infection risk at the early periods of treatment. Nevertheless, the risk attenuates significantly after the 2nd year of therapy. Clinicians should closely monitor infection status in patients with osteoporosis during the initial stages of denosumab therapy.

## Introduction

Osteoporosis is a progressive condition of bone fragility leading to risks of fracture. Denosumab, a fully human monoclonal antibody, binds with high specificity to the RANK ligand and reduces the number and activity of osteoclasts (OCs). In a 3-year randomized FREEDOM clinical trial, with a 7-year extension to evaluate efficacy and safety, denosumab demonstrated significant efficacy in reducing the incidence of hip, vertebral, and nonvertebral fractures of postmenopausal women with osteoporosis (1). Once age-related osteoporosis develops, patients will likely require lifelong treatment. This exposes patients to the adverse events of cumulative drug exposure, such as osteonecrosis of the jaw. Despite the benefits of anti-osteoporosis treatment, the risks of long-term exposure to osteoporosis drugs need to be evaluated, particularly in the older adult population (2).

The RANKL–RANK–OPG (osteoprotegerin) pathway involves the tumor necrosis factor (TNF) and TNF receptor superfamilies, which have many common signaling characteristics (3). The RANKL–RANK–OPG pathway was first discovered in the late 1990s and was considered important to immunity, primarily through its actions on dendritic cells (DCs) (4, 5). Concomitantly, it was determined to be critical to bone homeostasis through its regulation of OCs (6, 7). Denosumab (formerly AMG 162) is an effective compound that binds to and inhibits RANKL (8). Similar to OPG, denosumab inhibits bone loss by inhibiting RANKL’s action on the receptor RANK.

Reports concerning the relationship between denosumab and the risk of infection have been inconsistent. The relevant studies are limited by small sample sizes and short study periods. More importantly, infection risk was not the primary outcome in these studies. Accordingly, we conducted a population-based national cohort study to evaluate the infection risk in patients with osteoporosis after long-term denosumab treatment.

## Methods

### Data source

We used the Taiwan National Health Insurance Research Database (NHIRD), which is a valuable resource for public health research. The NHIRD contains comprehensive claims records related to outpatient and inpatient medical care, comprising the demographic information of beneficiaries; hospital care data; laboratory tests; diagnostic codes; interventional procedures; and drug prescription data such as drug quantity, expenditure, and dosage. All data are encrypted and deidentified to protect personal privacy. The diagnoses in the database are based on the *International Classification of Diseases, 9th Revision* and *10th Revision, Clinical Modification* (*ICD-9-CM* & *ICD-10-CM*, respectively). Our study was approved by the Institutional Review Board of China Medical University Hospital Research Ethics Committee (CMUH109-REC2-031[CR-2]), and was conducted in accordance with the guidelines of the World Medical Association’s Declaration of Helsinki.

### Study population

**Figure 1:**
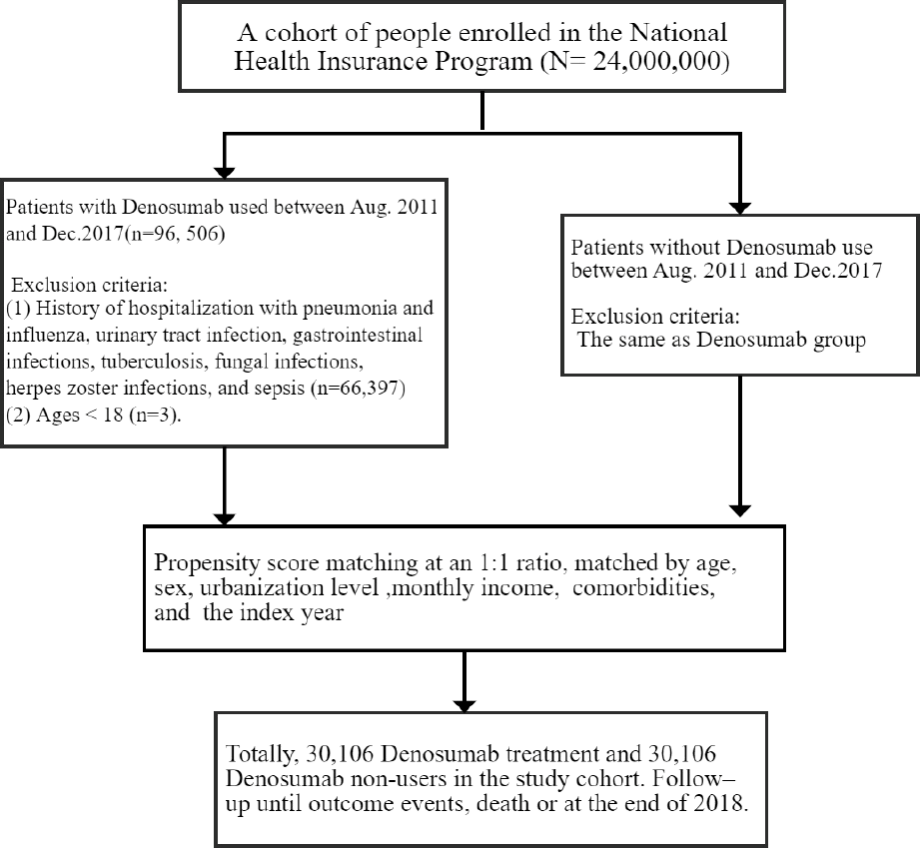
Participant selection flowchart.

This retrospective cohort study focused on patients with osteoporosis who had or had not received denosumab therapy. The case cohort comprised patients who were treated with denosumab. The index date was the first day of denosumab therapy. Denosumab users received a subcutaneous dose of 60 mg every 6 months depending on clinical responses. The control cohort comprised patients who had not received denosumab therapy; these patients were selected on the basis of propensity score (PS) matching for balancing heterogenous factors such as age, gender, index year, comorbidities, and other medicines, and they were assigned to the control cohort at a 1:1 ratio to the case patients. Each member of the control cohort was assigned a random index date that postdated their diagnosis of osteoporosis. The exclusion criteria for both cohorts were age <18 years and outcomes (namely, hospitalization for infection) before the index date. The enrollment period was between August 2011 and December 2017 (**Figure 1**).

### Outcome measurement and covariate

The study outcomes were the following infections: pneumonia and influenza (*ICD-9-CM* codes 480–488; *ICD-10-CM* code J09–J18); urinary tract infection (*ICD-9-CM* code 599; *ICD-10-CM* code N390); gastrointestinal infection (*ICD-9-CM* code 0088; *ICD-10-CM* codes A084, A088); tuberculosis (*ICD-9-CM* codes 010–018; *ICD-10-CM* codes A15–A19); fungal infection (*ICD-9-CM* codes 112, 1173, 1363, 3210, 4846, 51636, 5186; *ICD-10-CM* codes B37, B44, B45, B59, J84114), herpes zoster infection (*ICD-9-CM* codes 053, 054; *ICD-10-CM* codes B00, B02) and sepsis (*ICD-9-CM* code 99591; *ICD-10-CM* code A419). Patients were censored at the date of death, withdrawal from the health insurance system, or on Dec 31, 2018, whichever came first. Patients who died or left the program before the end of study were censored.

The following confounders were compared between cohorts: age, sex, urbanization level, monthly income, hypertension, type 2 diabetes mellitus (T2DM), hyperlipidemia, chronic obstructive pulmonary disease (COPD), obesity, cirrhosis, chronic kidney disease, heart failure, and fracture.

### Statistical analysis

Data are presented as percentages and mean values. We used the chi-square test to compare proportional differences in baseline variables between case and control patients. A Wilcoxon signed-rank test was used to compare the median ages between the two groups. Incidence rates are presented as the number of events of 1000 person–years. The Cox proportional hazard regression model was used to compare the risks of developing outcome events between case and control patients. Adjusted hazard ratios (HRs) and 95% confidence intervals (CIs) were calculated, with adjustment for important risk factors for developing study outcomes, including age, sex, urbanization level, monthly income, and comorbidities. We also considered death as a competing factor to estimate subhazard ratios (SHRs) and 95% confidence intervals (CIs) using the competing-risks regression models. The risk of study outcomes over time for patients who received denosumab therapy as compared with controls was determined using survival analysis with the Kaplan–Meier method, and the differences were evaluated using a log-rank test. All statistical analyses were performed using SAS (v.9.4; SAS Institute, Cary, NC), and R software (v.4.1.0, R Foundation, Vienna, Austria). Statistical significance was defined as a *p* value of <0.05.

## Results

A total of 30,106 pairs of PS-matched case and control patients were analyzed. In the denosumab cohort, the median age was 76 years, and the majority of participants were women (86.1%); 48% of the participants lived in high-urbanization areas, and 56% had a monthly income between NT$20,001 and NT$39,999. The prevalent comorbidities in the denosumab cohort were hypertension (72.1%), hyperlipidemia (54.1%), and COPD (28%). During the follow-up period, 69.5% of the participants in the denosumab cohort received <4 injections of denosumab, 69.5% received 5–8 injections, and 8.09% received ≥9 injections (**Table 1**).

**Table 1:** Demographic characteristics, comorbidities and medications in patients with osteoporosis with and without denosumab use.

	Denosumab		*p*-value
	No	Yes	
	(N =30106)	(N =30106)	
Variables	n (%)	n (%)	
Age stratified			0.59
≤ 49	162 (0.54)	179 (0.59)	
50-64	3912 (13.0)	3956 (13.1)	
≧65	26032 (86.5)	25971 (86.3)	
Age, median (IQR) ^a^	76 (70-82)	76 (69-82)	0.34
Sex			0.44
Female	25987 (86.3)	25922 (86.1)	
Male	4119 (13.7)	4184 (13.9)	
Urbanization level			0.99
1 (highest)	14458 (48.0)	14443 (48.0)	
2	12067 (40.1)	12068 (40.1)	
3	2706 (8.99)	2705 (8.98)	
4 (lowest)	875 (2.91)	890 (2.96)	
Monthly income (NTD)			0.98
<20,000	8522 (28.3)	8530 (28.3)	
20,001-39,999	16820 (55.9)	16800 (55.8)	
≥40,000	4764 (15.8)	4776 (15.9)	
Comorbidity			
Hypertension	21714 (72.1)	21700 (72.1)	0.90
T2DM	3723 (12.4)	3715 (12.3)	0.92
Hyperlipidemia	16326 (54.2)	16276 (54.1)	0.68
COPD	8425 (28.0)	8422 (28.0)	0.98
Obesity	342 (1.14)	391 (1.30)	0.07
Cirrhosis	6366 (21.2)	6374 (21.2)	0.94
Chronic kidney disease	2164 (7.19)	2153 (7.15)	0.86
Heart failure	2164 (7.19)	2153 (7.15)	0.86
Fracture	4476 (14.9)	10066 (33.4)	0.001
Prolia dosages in ampoule (Am.)			
≤4		20935 (69.5)	
5-8		6735 (22.4)	
≥9		2436 (8.09)	

Chi-square test; ^a^ Wilcoxon signed-rank test.

T2DM, type 2 diabetes metellus; COPD, chronic obstructive pulmonary disease.

The outcomes and incidence rates of infections and death are presented in **Table 2**. In the denosumab cohort, urinary tract infection had the highest incidence rate (113.6 per 1000 person–years), followed by pneumonia and influenza (54.6 per 1000 person–years), herpes zoster infection (21.7 per 1000 person–years), and serious adverse events of sepsis (18.4 per 1000 person–years). Compared with the control group, patients receiving denosumab therapy had significantly higher risks of pneumonia and influenza (adjusted HR [aHR]: 1.33; 95% confidence interval [CI]: 1.27–1.39), urinary tract infection (aHR: 1.36; 95% CI: 1.32–1.40), tuberculosis (aHR: 1.60; 95% CI: 1.36–1.87), fungal infection (aHR: 1.67; 95% CI: 1.46–1.90), candidiasis (aHR: 1.68; 95% CI: 1.47–1.93), herpes zoster infection (aHR: 1.27; 95% CI: 1.19–1.35), and sepsis (aHR: 1.54; 95% CI: 1.43–1.66). Additionally, patients receiving denosumab therapy had a 26% higher risk of death (aHR: 1.26; 95% CI: 1.20–1.32; *p* < 0.001) than controls. **Table 2** shows the subhazard ratios of outcome for patients receiving denosumab therapy, with consideration of the competing risk of death. We found that the denosumab cohort still remained a significantly higher risk of outcome than did in the control group, except for gastrointestinal infections.

**Table 2:** Clinical outcomes of patients with and without denosumab use.

	Denosumab									
	No (N=30106)			Yes (N=30106)						
Outcome	Event	PY	Rate^#^	Event	PY	Rate^#^	Crude HR (95% CI)	Adjusted HR^†^ (95% CI)	Crude SHR (95% CI)	Adjusted HR^†^ (95% CI)
Pneumonia and influenza	3794	91225	41.6	4784	87635	54.6	1.32(1.26, 1.38)***	1.33(1.27, 1.39)***	1.31(1.25, 1.36)***	1.25(1.20, 1.31)***
Urinary tract infection	7187	85359	84.2	9064	79768	113.6	1.36(1.32, 1.41)***	1.36(1.32, 1.40)***	1.34(1.30, 1.38)***	1.31(1.27, 1.36)***
Gastrointestinal infections	35	95213	0.37	32	92724	0.35	0.94(0.58, 1.52)	0.94(0.58, 1.52)	0.92(0.57, 1.49)	1.00(0.61, 1.65)
Tuberculosis	250	94896	2.63	384	92254	4.16	1.58(1.35, 1.85)***	1.60(1.36, 1.87)***	1.55(1.32, 1.82)***	1.49(1.26, 1.76)***
Fungal infections	350	94745	3.69	568	91863	6.18	1.67(1.46, 1.91)***	1.67(1.46, 1.90)***	1.64(1.44, 1.87)***	1.61(1.41, 1.85)***
Candidiasis	333		3.51	545		5.93	1.68(1.47, 1.93)***	1.68(1.47, 1.93)***	1.65(1.44, 1.90)***	1.62(1.41, 1.87)***
Herpes zoster infections	1577	92119	17.1	1932	88931	21.7	1.27(1.19, 1.36)***	1.27(1.19, 1.35)***	1.25(1.17, 1.33)***	1.25(1.16, 1.33)***
Sepsis	1141	94472	12.1	1686	91420	18.4	1.53(1.42, 1.65)***	1.54(1.43, 1.66)***	1.51(1.40, 1.63)***	1.44(1.33, 1.55)***
Death	3313	95266	34.8	4030	92786	43.4	1.25(1.20, 1.31)***	1.26(1.20, 1.32)***		

PY: person–year; Rate^#^, incidence rate, per 1,000 person–years; crude HR: relative hazard ratio;

Adjusted HR^†^: multivariable analysis including age, sex, urbanization level, monthly income and comorbidities.

***p < 0.001.

**Figure 2:**
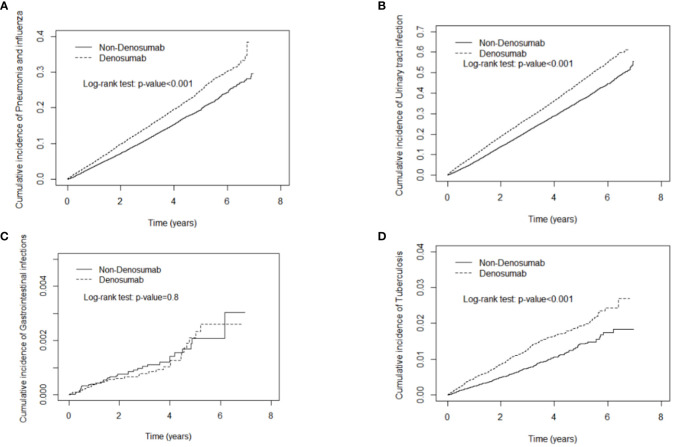
Cumulative incidence curves of pneumonia and influenza **(A)**, urinary tract infection **(B)**, gastrointestinal infections **(C)**, tuberculosis **(D)** in patients with and without denosumab use.

**Figure 3:**
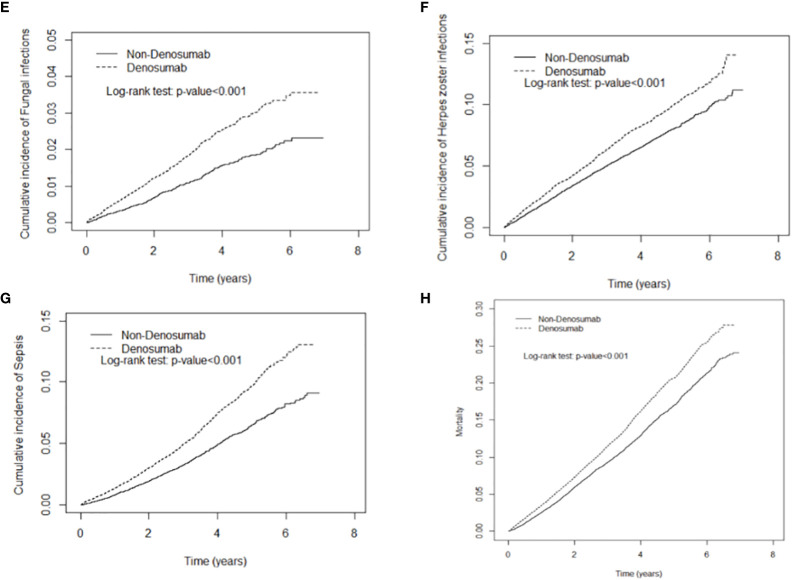
Cumulative incidence curves of fungal infection **(E)**, herpes zoster infection **(F)**, sepsis **(G)**, mortality **(H)** in patients with and without denosumab use.

Denosumab use was not significantly associated with gastrointestinal infection. As demonstrated in **Figures 2**, **3**, the cumulative risks of infection and death in the denosumab cohort were significantly higher than those in the control cohort in terms of pneumonia and influenza, urinary tract infection, tuberculosis, fungal infection, herpes zoster infection, and sepsis, with a follow-up duration longer than 6 years.

**Table 3** displays the dose effect of denosumab on infectious outcomes. Compared with the control cohort, denosumab users receiving ≤4 cumulative doses had significantly higher risks of pneumonia and influenza (aHR: 1.85; 95% CI: 1.77–1.94), urinary tract infection (aHR: 1.66; 95% CI: 1.60–1.71), tuberculosis (aHR: 2.23; 95% CI: 1.89–2.63), fungal infection (aHR: 2.38; 95% CI: 2.07–2.73), herpes zoster infection (aHR: 1.74; 95% CI: 1.63–1.88), and sepsis (aHR: 2.10; 95% CI: 1.94–2.27). However, after cumulative denosumab use exceeded 5 doses, higher cumulative doses were associated with a lower risk of certain infections. Compared with controls, patients who received 5–8 injections of denosumab had a 28% lower risk of pneumonia and influenza (aHR: 0.72; 95% CI: 0.66–0.78), and those receiving ≥9 injections had a 73% lower risk (aHR: 0.27; 95% CI: 0.230–0.32). The association between higher cumulative doses of denosumab and lower risk of infection was also observed for tuberculosis, fungal infection, and herpes zoster infection. The consistent results were shown in the competing-risks regression models.

**Table 3:** Outcomes related to denosumab dosage in patients with and without denosumab use.

Outcome	Event	PY	Rate^#^	Crude HR (95% CI)	Adjusted HR^†^ (95% CI)	Crude SHR (95% CI)	Adjusted SHR^†^ (95% CI)
Pneumonia and influenza							
Prolia dosages (Amp.)							
Non-users	3794	91225	41.6	Ref.	Ref.	Ref.	Ref.
<=4	3958	50944	77.7	1.94 (1.85, 2.03)***	1.85 (1.77, 1.94)***	1.85 (1.77, 1.93)***	1.67 (1.60, 1.75)***
5-8	686	24044	28.5	0.69 (0.63, 0.75)***	0.72 (0.66, 0.78)***	0.70 (0.65, 0.76)***	0.71 (0.66, 0.77)***
>=9	140	12647	11.1	0.24 (0.20, 0.29)***	0.27 (0.23, 0.32)***	0.26 (0.22, 0.30)***	0.29 (0.25, 0.34)***
Urinary tract infection							
Prolia dosages (Amp.)							
Non-users	7187	85359	84.2	Ref.	Ref.	Ref.	Ref.
<=4	6358	46896	135.6	1.68 (1.62, 1.74)***	1.66 (1.60, 1.71)***	1.58 (1.53, 1.63)***	1.53 (1.48, 1.59)***
5-8	1944	21724	89.5	1.07 (1.02, 1.13)**	1.08 (1.02, 1.13)**	1.10 (1.05, 1.16)***	1.08 (1.03, 1.14)***
>=9	762	11148	68.4	0.74 (0.69, 0.80)***	0.76 (0.70, 0.82)***	0.80 (0.74, 0.86)***	0.80 (0.75, 0.86)***
Tuberculosis							
Prolia dosages (Amp.)							
Non-users	250	94896	2.63	Ref.	Ref.	Ref.	Ref.
<=4	338	54994	6.15	2.36 (2.00, 2.78)***	2.23 (1.89, 2.63)***	1.07 (0.63, 1.81)	1.17 (0.67, 2.03)
5-8	38	24549	1.55	0.59 (0.42, 0.82)**	0.62 (0.44, 0.88)**	0.84 (0.37, 1.88)	0.90 (0.39, 2.08)
>=9	8	12711	0.63	0.23 (0.11, 0.47)***	0.28 (0.14, 0.58)***	0.42 (0.10, 1.68)	0.44 (0.11, 1.78)
Fungal infections							
Prolia dosages (Amp.)							
Non-users	350	94745	3.69	Ref.	Ref.	Ref.	Ref.
<=4	483	54675	8.83	2.42 (2.11, 2.78)***	2.38 (2.07, 2.73)***	2.21 (1.87, 2.59)***	1.97 (1.67, 2.34)***
5-8	76	24478	3.10	0.83 (0.65, 1.06)	0.83 (0.65, 1.07)	0.61 (0.43, 0.85)**	0.63 (0.45, 0.89)***
>=9	9	12711	0.71	0.19 (0.10, 0.36)***	0.19 (0.10, 0.38)***	0.26 (0.13, 0.51)***	0.31 (0.16, 0.63)***
Herpes zoster infections							
Prolia dosages (Amp.)							
Non-users	1577	92119	17.1	Ref.	Ref.	Ref.	Ref.
<=4	1547	52151	29.7	1.74 (1.62, 1.87)***	1.74 (1.63, 1.88)***	2.26 (1.97, 2.59)***	2.57 (2.03, 3.27)***
5-8	330	24107	13.7	0.80 (0.71, 0.90)***	0.79 (0.71, 0.90)***	0.86 (0.67, 1.10)	0.39 (0.31, 0.49)***
>=9	55	12673	4.34	0.25 (0.19, 0.33)***	0.25 (0.19, 0.32)***	0.20 (0.11, 0.39)***	0.50 (0.48, 0.52)***
Sepsis							
Prolia dosages (Amp.)							
Non-users	1141	94471	12.1	Ref.	Ref.	Ref.	Ref.
<=4	1391	54315	25.6	2.24 (2.07, 2.42)***	2.10 (1.94, 2.27)***	2.28 (1.98, 2.62)***	2.21 (1.91, 2.56)***
5-8	255	24409	10.5	0.86 (0.75, 0.98)*	0.91 (0.80, 1.05)	0.86 (0.67, 1.10)	0.85 (0.66, 1.10)
>=9	40	12696	3.15	0.22 (0.16, 0.31)***	0.26 (0.19, 0.36)***	0.22 (0.11, 0.41)***	0.22 (0.11, 0.42)***

PY: person–year; Rate^#^, incidence rate, per 1,000 person–years; crude HR, relative hazard ratio;

Ref: Reference group

Adjusted HR^†^: multivariable analysis including age, sex, urbanization level, monthly income and comorbidities.

*, p < 0.05; **, p < 0.01; ***, p < 0.001.

## Discussion

In this large-scale 10-year observational study of 30,160 patients receiving denosumab therapy, infection risks were investigated as the primary outcome. After adjustment for confounders including diabetes, chronic kidney disease, obesity, liver cirrhosis, chronic pulmonary disease, heart failure and fracture, the multivariate analysis revealed a significantly higher infection rate in patients with osteoporosis who received denosumab treatment than in those who did not. Additionally, we compared patient survival rates and observed a 1.26-fold higher risk of death in patients treated with denosumab compared with controls. A meta-analysis of 22,253 patients who received denosumab therapy revealed a 1.21-fold increase in severe adverse events infection (SAEI) compared with controls (9). Our findings are consistent with the aforementioned result.

The present study investigated the rates of infections, including pneumonia and influenza, urinary tract infection, tuberculosis, candidiasis, fungal infection (*Aspergillosis* or *Cryptococcosis)*, herpes zoster infection, and sepsis, in the denosumab cohort and the control cohort. Intergroup comparisons revealed a consistent trend across the various infections: denosumab treatment was significantly associated with a higher infection risk in patients with osteoporosis. Specifically, we found a 1.60-fold higher risk of tuberculosis infection, 1.67-fold higher risk of fungal infection, 1.68-fold higher risk of candidiasis infection, and 1.27-fold higher risk of herpes zoster infection. To investigate whether mortality was associated with the effect of denosumab on infection risks, competing risk analysis with mortality was done as well. Our findings revealed that consistent results regarding the effect of denosumab on infection risks were observed in patients with osteoporosis. To the best of our knowledge, the current study is the first to report on the higher risks of infections in patients receiving denosumab treatment.

Substantial evidence suggests that RANK or RANKL mRNA expression is detected in immune tissues apart from osteoblasts, osteocytes, and bone stroma. For example, RANK expression has been identified in immune cells including T lymphocytes and B cells in the lung (6, 7, 10, 11). RANK expression is predominant in mature DCs. DCs are believed to share a common lineage with OCs and macrophages (4, 5). T cell activation *via* major histocompatibility complex and antigen–TCR interactions is likely mediated by DCs (3). RANKL expression plays a critical role in T cell function and presumably promotes T cell activation, which is dependent on its action on DCs. This in turn strengthens the survival of DCs in addition to triggering a mutual immune reaction. The subsequent engagement of T cells with DCs induces the differentiation of subsets of T cells for their transformation into Th1, Th2, and Th17 cells (3). Although RANKL inhibits bone metabolism, it is unclear whether RANKL inhibition might also attenuate the systemic immune response.

No consistent results on this issue related to the immune response (mentioned above) have been presented thus far. A previous study reported that in some specific tissues such as the skin, the RANKL–RANK interaction may alter the intensity of the inflammatory reaction beyond an immunosuppression response (12, 13). By contrast, a relevant study explored the differences between RANKL pathway inhibition and the complete absence of RANK or RANKL expression. The findings in an animal model revealed that the inhibition of RANKL could reduce bone loss by increasing bone density, but with no effect on inflammatory reactions (14). Our key finding is that denosumab use is significantly associated with a higher infection risk, including tuberculosis, candidiasis, herpes zoster virus, and fungal infection. This finding suggests that a modest attenuated cellular immunity response occurs in patients with osteoporosis under denosumab treatment.

Additionally, we investigated the immunosuppressive effects of cumulative denosumab dosages on infection risk in separate time periods. We observed that the infection risk was significantly higher after the first 4 consecutive doses of denosumab within 2 years; however, the risk attenuated thereafter. To our knowledge, it is the first study to disclose the reciprocal relationship between infection risk in osteoporosis patients and the immunosuppression nature by accumulative dosage of denosumab. The finding was novel which has never been identified previously.

The reasons to explain the infection risk and transient immunosuppressive effect of denosumab need to be elucidated further. In certain conditions, a high infection risk post-transplantation developed in new organ transplant recipients who received immunosuppression initially but the risk declined gradually in the later period post-transplantation, which was similar to our findings in this study to some degree. For example, a previous study reported that the incidence rate of herpes virus infection was highest in the first year after transplantation but the risk attenuated thereafter, which suggests that acute impairment of T cellular immunity occurs in the first several months following transplantation (15).

Altogether, we hypothesize that the acute immunosuppressive effect of denosumab developed initially but lasted transiently. More evidence is needed to investigate the sophisticated mechanism in the future.

## Limitations

Our study results should be interpreted with caution due to some inherent limitations. First, information on lifestyle, body mass index (or obesity) and personal behaviors (such as smoking and alcoholic drinking) is not available from the NHIRD; therefore, we applied proxy variables for comorbidities. For example, the COPD variable was replaced by smoking to attenuate the confounding effect. Second, our data were retrieved from an inpatient database, meaning that only instances of severe infection were analyzed in this study. Patients with infectious diseases of mild severity may have been overlooked; thus, the actual risks of infections were likely underestimated for the denosumab cohort. Third, lack of individual laboratory data such as circulating 25(OH) D levels and imaging finding in the NHIRD may be the other study limitation.

Finally, despite our best attempts to control for all possible disease-associated confounders, there are still many unknown or uncontrolled confounding factors and some inherent bias may have affected the study findings. The retrospective study is usually lower evidence than the randomized controlled trials. Such shortcoming can be overcome by conducting a randomized control trial in the future.

## Conclusion

Our findings provide evidence that denosumab use is associated with a high risk of opportunistic infection during the first 2 years of administration. Based on the findings, the effects of immunosuppression associated with denosumab use may be attributed to RANKL inhibition.

## Data availability statement

The original contributions presented in the study are included in the article/supplementary materials. Further inquiries can be directed to the corresponding authors.

## Ethics statement

Our study was approved by the Institutional Review Board of China Medical University Hospital Research Ethics Committee (CMUH109-REC2-031[CR-2]), and was conducted in accordance with the guidelines of the World Medical Association’s Declaration of Helsinki. Written informed consent for participation was not required for this study in accordance with the national legislation and the institutional requirements.

## Author contributions

All authors have contributed significantly, and that all authors are in agreement with the content of the manuscript: Conception/Design: S-TH, T-MY. Provision of study materials: C-LL, C-HK. Collection and/or assembly of data: C-LL. Data analysis and interpretation: all authors. Manuscript writing: all authors. Final approval of manuscript: all authors.
